# Nutritional Traits, Pasting Properties and Antioxidant Profile of Selected Genotypes of Sorghum, Oat and Maize Eligible for Gluten-Free Products

**DOI:** 10.3390/foods13070990

**Published:** 2024-03-24

**Authors:** Laura Gazza, Valeria Menga, Federica Taddei, Francesca Nocente, Elena Galassi, Chiara Natale, Chiara Lanzanova, Silvana Paone, Clara Fares

**Affiliations:** 1 CREA-IT Consiglio Per la Ricerca in Agricoltura e L’analisi Dell’economia Agraria Centro di Ricerca Ingegneria e Trasformazioni Agroalimentari, Via Manziana, 30, 00189 Roma, Italy; laura.gazza@crea.gov.it (L.G.); federica.taddei@crea.gov.it (F.T.); francesca.nocente@crea.gov.it (F.N.); elena.galassi@crea.gov.it (E.G.); chiara.natale@crea.gov.it (C.N.); 2 CREA-CI Consiglio Per la Ricerca in Agricoltura e L’analisi Dell’economia Agraria Centro di Ricerca Cerealicoltura e Colture Industriali, S.S. 673, km 25.200, 71122 Foggia, Italy; valeria.menga@crea.gov.it (V.M.); silvana.paone@crea.gov.it (S.P.); 3 CREA-CI Consiglio Per la Ricerca in Agricoltura e L’analisi Dell’economia Agraria Centro di Ricerca Cerealicoltura e Colture Industriali, Via Stezzano, 24, 24126 Bergamo, Italy; chiara.lanzanova@crea.gov.it

**Keywords:** gluten-free, pasting properties, antioxidant activity, pigmented maize, food grade sorghum, oat

## Abstract

The technological and nutritional traits of food-grade sorghum hybrids, hulled/naked oat varieties and maize genotypes of different colors were studied for novel and healthier gluten-free foods. Oat genotypes showed the highest protein content, followed by maize and sorghum. The total starch and the total dietary fiber content were quite similar among the three species. Great variation was found in the amylose content, and the highest was in sorghum (27.12%), followed by oat 16.71% and maize 10.59%. Regarding the pasting profile, the rank of Peak Viscosity was sorghum (742.8 Brabender Unit, BU), followed by maize (729.3 BU) and oat (685.9 BU). Oat and sorghum genotypes had similar average breakdown (407.7 and 419.9 BU, respectively) and setback (690.7 and 682.1 BU, respectively), whereas maize showed lower values for both parameters (384.1 BU and 616.2 BU, respectively). The total antioxidant capacity, only in maize, significantly correlated with total flavonoid, phenolic and proanthocyanidin contents, indicating that all the measured compounds contributed to antioxidant capacity. The study indicated the importance of sounding out the nutritional and technological characteristics of gluten-free cereals in order to select suitable cultivars to be processed in different gluten-free foods with better and healthier quality.

## 1. Introduction

In the last 50 years, an increasing trend in the incidence of coeliac disease (CD) has been observed, but at present, a lifelong, strict gluten-free diet is the only effective treatment. However, lifelong gluten exclusion from the diet may have a negative impact on the quality of life of CD patients [1]. Furthermore, nutritional complications are frequent, as gluten-free food is often rich in lipids and poor in vitamins, antioxidants and fibers [2]. There is an increasing interest in the search for gluten-free cereal species with good baking performance suitable for the diet of CD patients [3], but insufficient information is available to identify genotypes for obtaining gluten-free pasta of good acceptability and technological quality [4]. The rheological properties of doughs could be used as a tool to select appropriate raw materials and investigate their behavior under different transformation processes in terms of suitable product formulation. Among the gluten-free cereal species, sorghum, oat and maize represent valuable raw materials for the development of products with improved nutritional value.

Sorghum (*Sorghum bicolor* L.) is the fifth cereal crop worldwide, and it is considered among the species that could ensure global food security in the climate change scenario. Besides its easy adaptability to a wide range of growing conditions, being a C4 plant, sorghum has higher photosynthetic nitrogen- and water-use efficiencies than C3 plants, hence good tolerance to drought, which is expected to increase in the future. Sorghum, like other cereals, is an excellent source of starch and proteins. It represents the major staple food crop for a fair share of the populations living in the semi-arid tropics [5], whereas in Western countries, it is mainly intended for feed and ethanol production. In recent years, the interest in sorghum for human foods has increased due to its nutritional characteristics, such as the presence of bioactive compounds (especially flavonoids), oligo-elements (mainly magnesium and manganese) and fatty acid profile (omega 6 and 3). Moreover, the low glycaemic index and the absence of gluten make it beneficial to diabetics and safe for people affected by celiac disease or gluten intolerances [6]. Around the world, it is consumed as porridges, bread, cookies, tortillas, extruded products and beverages [7], and in recent years it has been processed into noodles and pasta [8,9].

Oat (*Avena sativa* L.) is the seventh cereal crop worldwide, but with a general decreasing trend [10]; in particular, in Western Europe, the area dedicated to oat has halved in the last 30 years. Oat is rich in substances beneficial to the human body, vitamin E (tocols), phytic acid, phenolic compounds and avenanthramides are the most abundant antioxidants, but flavonoids and sterols are also present. These compounds are concentrated in the outer layers of the seed [11]. The introduction of oats in the formulation of GF products is very recent, and the AIC (Associazione Italiana Celiachia) [12] has fully included it in the gluten-free cereals. In addition to the antioxidants listed above, the richness of beta-glucans and insoluble fiber represent functional compounds that, in the formulation of GF products, can make a significant contribution to improving the health quality of these foods. Several studies have indicated that the prevalence of celiac disease in patients with type 1 diabetes mellitus (T1DM) is between 3 and 10%, which is more than ten times the prevalence in the general population [13]. Both diseases result from a complex interaction between genetic susceptibility and environmental exposure, being associated with the major histocompatibility complex class II antigen DQ2 and sharing non-HLA loci [14]. These insights make the need to develop low glycemic index foods more compelling.

Maize (*Zea mays* L.) is one of the most important crops cultivated worldwide due to its huge versatility and multiple uses such as food, forage and industrial purposes. In Latin America, Asia and Africa, it is used for the preparation of traditional foods [15], but in recent years, in Western countries, the use of this crop for gluten-free foods has increased due to the increase in consumption of GF foods. Maize is a good source of starch, proteins and lipids, and it also contains several bioactive compounds that are important for human health [16]. The different types of maize are characterized by a grain color varying from white to yellow, pink, red, blue or black. Pigmented maize contains many secondary metabolites, such as phenolic compounds, anthocyanins, carotenoids and tocols [17,18]. The principal distinction between blue and purple maize varieties is the different localization in the distribution of anthocyanins. In purple maize varieties, these flavonoids are localized most abundantly in the pericarp of the kernel, while blue varieties typically produce anthocyanins in the aleurone [19]. Because the content of proteins and antioxidants in traditional GF flours is low, the use of nutrient and dietary fiber-rich ingredients has been proposed to improve the nutritional quality of such foods [20]. Therefore, the aim of this study was to analyze the technological, biochemical, and nutritional characteristics of twenty-six genotypes of food-grade sorghum, oat and maize in order to find eligible genotypes for developing novel and healthier gluten-free products.

## 2. Materials and Methods

### 2.1. Plant Material

Seeds and flours of food grade/zootechnical use hybrids of sorghum, genotypes of naked/hulled seed of oat with both clear and colored glumes, and white/pigmented maize genotypes, were analyzed.

Oat, maize and sorghum genotypes were derived from seed collections of CREA Research Centre for Cereal and Industrial Crops, Foggia and Bergamo and Research Centre for Engineering and Agro-food Processing of Rome, respectively. In particular, were analyzed: (i) one sorghum genotype for zootechnical use (cultivar Aralba, RV-Venturoli, Bologna, Italy), four food-grade hybrids PSE7431, PSEAG4E44, DMS (Padana Sementi Elette, Padova, Italy), Diamond (APSOV Sementi, Voghera, PV, Italy), and three Bolivian food-grade hybrids SW6129, SW6143W, SW6237W (SemWest Semillas, LaPaz Bolivia); (ii) six naked oat genotypes (Abel, Konradin, Kripton, Kynom, Lexic and Terra) and three hulled genotypes (Corneil, Genziana and Nigra); (iii) two maize Bolivian landraces, Hualtaco (white) and Kully (purple), collected in the frame of International Cooperation Project (P.S.G.O. km0 Bolivia), four Italian landraces VA116W and VA522W (white) VA 572 (yellow), VA1268 (red), and three pigmented genotypes VA116 (purple), VA522 (blue) and VA572 (black), the latter three obtained by crossing VA116W, VA522W, VA572 with germplasm of Bolivian origin “Kully”, Mexican “Azul” and Spanish “Millo Corvo”.

### 2.2. Chemical and Physical Characterization

All samples were milled to whole meal flour using a laboratory mill (Retsch ZM 200 Haan, Dusseldorf, Germany) at 12,000 rpm and 0.5 or 1.0-mm sieve, depending on the requirements of each analysis. All analyses were performed in triplicate. The sample moisture was measured using a thermobalance (Sartorius MA 40, Goettingen, Germany) at 120 °C just before the chemical analyses in order to express all data on a dry weight basis (dw). Protein content was measured according to the ICC 105/2 method [21]. Factors of 6.25 for sorghum and maize and 5.83 for oats were used to convert nitrogen to protein [22]. Total and resistant starch (TS and RS) content were determined by enzymatic method using the Megazyme (Bray, Dublin, Ireland) kits K-TSTA and K-RSTAR according to AOAC methods 996.11 [23] and 2002.02 [24], respectively. Amylose content was determined using the Megazyme Amylose/Amylopectin assay kit K-AMYL. The content of total dietary fiber (TDF) was measured using an enzymatic kit for fiber determination (Bioquant, Merck, Darmstadt, Germany) according to the AOAC Official Method 991.42 [25]. Protein, TS, RS, TDF and Amylose content were expressed as percentage *w*/*w* on dry weight basis. The methods ISO 520:2010 [26] and ISO 7971-1:2009 [27] were used to determine the thousand kernel weight (TKW) and test weight (TW). The β-glucan content was determined by enzymatic method according to AOAC Method 995.16 [28].

### 2.3. Total Flavonoid, Phenolic, Proanthocyanidin Contents and Antioxidant Capacities

Phenolic compounds were extracted according to Beta et al. [29], with minor modifications. The samples (1 g), ground with a 1.0 mm– sieve, were extracted using 8 mL methanol acidified with 1N HCl (80:20; *v*/*v*) for 30 min in an ultrasonic bath (BRANSONIC 2200, Branson, Danbury, NY, USA). The mixtures were centrifuged at 1000× *g* for 15 min and were used for the determination of the total phenolic content (TPC), total proanthocyanidin content (TPAC), total flavonoid content (TFC) and total antioxidant capacity (TAC) by ABTS assay.

Determination of total phenolic content was performed according to the procedure described by Singleton and Rossi [30]. Ferulic acid was used as the standard, and the data were expressed in mg ferulic acid equivalents (FA) per g of dry matter. A calibration curve was prepared by using increasing concentrations of ferulic acid (Sigma-Aldrich, Milano, Italy).

The total proanthocyanidin content (TPAC) was determined according to the modified vanillin assay [31] and expressed in mg catechin equivalents (CE) per g of dry matter.

The total flavonoid content (TFC) was determined according to Eberhardt et al. [32], using catechin as standard. Data were expressed in mg catechin equivalents (CE) per g of dry matter.

The measure of total antioxidant capacity (TAC) was determined according to Re et al. [33], with some modifications. ABTS (2,2-azino-bis-[3-ethylbenzothiazoline 6-sulphonic acid]) was dissolved in water at a 7 mM concentration. ABTS radical cation was produced, allowing the reaction of the ABTS stock solution with potassium persulfate (2.45 mM, final concentration) for 16 h in the dark and at room temperature before use. ABTS radical cation solution was diluted with ethanol to an absorbance of 0.70 ± 0.02 at 734 nm. The antioxidant capacity was expressed as Trolox equivalent antioxidant capacity (TEAC) in mmol of Trolox equivalents (TE)/kg sample on a dry matter basis.

### 2.4. Micro-Visco Amylograph Analysis

Pasting properties of the finely ground cereal samples (<0.5 mm) were measured using a micro visco-amylograph (Brabender OHG, Duisburg, Germany) according to Marengo et al. [34] with slight modifications. Fifteen grams (on a 14% moisture basis) of oat, maize or sorghum were suspended in 100 mL of distilled water and heated in the visco-amylograph by using the following time-temperature profile: heating from 30 up to 95 °C at a rate of 3 °C/min, holding at 95 °C for 20 min, cooling to 30 °C at a rate of 3 °C/min under constant stirring (250 rpm). The torque measuring range was 300 cmg. The viscosity was expressed in Brabender units (BU). The parameters resulting from this analysis were: pasting temperature (PT), defined as the minimum temperature for cooking the flour; peak viscosity (PV), defined as the highest viscosity reached during heating; peak temperature reached at PV, breakdown (BD), which represents the difference between the viscosity at the peak and at the minimum, setback, defined as the difference between the viscosity at the end of cooling period and the minimum viscosity at the start of cooling period.

### 2.5. Statistical Analyses

Replicated results were expressed as mean ± standard deviation. A one-way analysis of variance was performed with the MSTATC program (Michigan State University, East Lansing, MI, USA), followed by the Duncan multiple range test for a post-hoc comparison of means, applied to assess significant differences (*p* ≤ 0.05) for each considered parameter. Correlation analysis between the parameters was performed by using Past software (4.03 free version) through Pearson’s r coefficient (*p* ≤ 0.05).

## 3. Results and Discussion

### 3.1. Physical and Nutritional Traits

#### 3.1.1. Thousand Kernel Weight

Thousand kernel weight (TKW, Table 1) is an important technological parameter, playing a large role in flour yield at milling [35]. Little variability was found amongst sorghum hybrids, with a mean value of 23.26 g; the minimum and the maximum values were observed in PSE7431 (21.30 g) and DMS (25.62 g), respectively. These values were similar to those reported by Galassi et al. [36] and fell in the range found by Aruna et al. [37], who analyzed 60 sorghum genotypes amongst germplasm, cultivated and parental lines of the hybrids. Oat genotypes exhibited TKW values ranging from 23.70 g (Genziana, hulled genotype) to 12.55 g (Kripton, naked genotype); the average TWK was 16.49 g and 21.78 g for naked and hulled genotypes, respectively. This variability was partially ascribable to the presence of husks in hulled genotypes but also to the minus kernel weight, typically of naked genotypes, as ascertained also by Buerstmayr et al. [38] in a large collection of 120 oat genotypes. Maize genotypes showed great variability in TKW values, ranging from a minimum of 246.0 g (VA116W) to a maximum of 741.5 g (Hualtaco), with an average of 375.7 g.

#### 3.1.2. Proteins

Besides their nutritional properties, proteins are important for the processing aptitude of cereals as they contribute, with the amylose and amylopectin chains, to giving a better texture to gluten-free products [39,40]. Among the three species analyzed, oat genotypes showed, on average, the highest protein content (14.07%), ranging from 13.18% to 17.00% observed in Genziana and Corneil, respectively (Table 1). Maize exhibited a mean value of 10.70% and ranged from 6.93% to 13.35% in Hualtaco and VA116 purple, respectively. Sorghum hybrids presented a mean value of 10.51% and ranged from 9.20% to 12.68% in PSE7431 and Diamond, respectively. It is also noteworthy that maize hybrids revealed huger variability when compared to both sorghum hybrids and oat genotypes for this parameter.

#### 3.1.3. Total Dietary Fiber and β-Glucans

High dietary fiber intake is associated with health benefits for the intestine, and fiber content should be taken into the right consideration in gluten-free cereals alternative to the more diffused refined rice (average TDF content reported in literature is 0.5%) for the manufacturing of gluten-free foods, since a long-term food choice of individuals following a strict gluten-free diet often resolved in not sufficient fiber intake, anyway generally under the recommended daily allowance (RDA = 25 g/die for an adult) [41]. On average, total dietary fiber content was quite similar amongst the three cereal species (Table 1), but a fair variability was observed, especially in sorghum hybrids and oat genotypes. Moreover, the high content of TDF in our samples of maize could be ascribable to the milling process adopted in our study, i.e., whole grain, and it was comparable with the study performed on whole grain maize by Blandino et al. (2017), who found about 11% TDF in their genotypes [42]. It should be noted that in oats, a certain amount of β-glucan also contributes to the TDF content. Indeed, β-glucan is a component of the soluble fiber, which plays a major role in a number of the putative health benefits attributed to oat and barley products, mainly the reduction of blood glucose and -cholesterol [43]. In addition to their health-promoting properties, β-glucans are used as thickeners, emulsifiers and fat substitutes in foods, which is of crucial importance mainly in the manufacturing of GF products. The β-glucan mean content was 3.45%, and the highest significant content was measured in Terra (4.80% naked genotype) and Corneil (4.63% hulled genotype). In maize and sorghum, the level of β-glucan was under the limit of the detection of the method.

#### 3.1.4. Starch Characterization

Maize and sorghum hybrids had similar total starch contents (TS), 68.69% on average, about 10 percentage points more than oat genotypes (Table 1); in particular, the highest TS content (74.88%) was detected in the SW6237W sorghum hybrid, whereas the lowest was recorded in oat cultivar Abel (51.65%). The high total starch content of sorghum and maize hybrids makes these cereals a valuable alternative raw material to produce highly nutritious gluten-free foods or for the extraction of starch and derivatives for industrial purposes.

Because of the health benefits related to foods with increased resistant starch (RS) and decreased glycemic index, there is a growing interest in developing foods with higher resistant starch contents, even if a very high amount of resistant starch could affect the digestibility of foods and intestinal functions [44]. RS content in all the gluten-free cereals analyzed was found below the quantification limit of the method (2%). Nevertheless, sorghum exhibited the highest average content (1.04%), followed by oat (0.50%) and maize, with very low values (0.18%). The highest content of RS was observed in the Bolivian SW sorghum samples, with an average of almost 2% (Table 1).

The same trend was observed for the amylose content, the highest values being reported in sorghum hybrids, on average 27.12%, followed by oat (16.71%) and maize (10.59%), confirming the positive correlation between RS and amylose content, as previously observed by Biselli et al. [45] in rice. 

#### 3.1.5. Pasting Properties

Starch functionality in foods is determined by amylose and amylopectin and the physical organization of these macromolecules into the granular structure [46]. Amylopectin, with its multiple branched chains of (1–4)-α-glucans interlinked by (1–6)-α-linkages, is the major component of starch, with the unbranched amylose accounting for the minor fraction. These two components have different solubility, with amylopectin being more soluble than amylose [47]. The micro-visco amylograph analysis mimics the cooking process and, thus, allows the measurement of the pasting properties of a flour-water mixture during continuous heating and mixing for a given period. The pasting profile provides a very important indication of the structural changes affecting the starch granules during gelatinization and retrogradation.

Sorghum had the highest mean value of the pasting temperature (PT) (Table 2) (74.1 °C), followed by maize (71.5 °C) and oat (60.1 °C); consequently, oat flours required less energy to cook than the other two species. Generally, lower PT relates to a lower degree of swelling of starch granules and lower starch content [48]. Observing the rank obtained, the lowest value of oat is found as this species showed the lowest starch content (TS), counterbalanced with the highest protein content (Table 1). The rank of the tree species of the mean value for the peak viscosity was the same as observed for PT (Table 2). The highest mean value was recorded in sorghum at 742.8 (BU), followed by maize at 729.3 (BU) and oat at 685.9 (BU). Among sorghum genotypes, a great variability was found, and Diamond, SW6129 and SW6237W showed the highest significant values, more than 800 BU, while PSE7431 showed the lowest value (512.5 BU). High peak viscosity is recognized as the marker of starch gelatinization intensity [49]. Therefore, SW6129 and SW6237W hybrids that have the highest PV and setback could be appreciable for food products that request stable thickening after heating and cooling, like in sauces production. On the contrary, PSEAG4E44, with low PV and setback, is more suitable for baking goods because the gel is not too hard and less inclined to water separation, thus having a longer shelf life (i.e., less staling). The mean value of viscosity of the breakdown was 419.9 BU, and six genotypes showed no statistical difference; conversely, the highest value was found in PSEAG4E44 (505 BU), and the lowest was in PSE7431 (350.5 BU). The breakdown provides information about the rigidity or fragility of swollen starch granules, and it is an index of paste stability during cooking. Accordingly, out of 7 sorghum genotypes analyzed, six had similar starch resistance to mechanical and thermal stresses, and only PSEAG4E44 had different behavior, confirming good pasting properties.

In oat, high variability was found for PV and Genziana and Nigra (hulled genotypes) had the highest value (932.5 and 782.5 BU, respectively). Among the naked genotypes, Kripton, Kynom and Terra recorded the best values (752.0, 683.0 and 687.5 BU, respectively). Despite the highest mean value of the breakdown (407.7 BU), a narrow variability among genotypes was found, and the highest value was in Genziana (548 BU). Setback indicated that the highest value was for Terra and Lexic (798.5 and 802.0 BU, respectively). Accordingly, Genziana could be used for sauce because of its high hot viscosity and cold viscosity. Terra and Lexic could be interesting for GF pasta as the high value of setback. Conversely, Kripton, with a lower retrogradation degree, could be more apt for baking goods. In maize, great variability among genotypes was observed for pasting temperature and the highest significant value was found for the genotype VA 572 (74.5 °C) and the lowest for Kully and Hualtaco (67.25 and 67.15 °C, respectively). Regarding the PV, high variability was found, and the best values were found for Hualtaco, which showed the highest PV with respect to all the genotypes of the three species (1204.5 BU). This behavior indicates that Hualtaco, a white genotype of Andean origin, requires a short time for cooking and reaches a high viscosity with respect to all other genotypes of this study, but with less stable starch (high BD) and thus, not suitable for food preparation. The maize mean value of the breakdown was 384.1 BU and was the lowest in comparison to oat and sorghum. Generally, a low value of BD is associated with great stability of starch granules and a decrease in the rate of collapsing of starch granules. In this view, VA572 black could be interesting because of its low breakdown and setback and because it is suitable for baking goods.

#### 3.1.6. Antioxidant Profile

Amongst the species analyzed, sorghum hybrids showed the lowest total flavonoid content (TFC), with an average value of 0.44 mgCE/g (Table 3). The highest and the lowest content occurred in PSE7431 and SW6129 (Bolivian genotype), respectively, with an average value of 0.85 mgCE/g and 0.31 mgCE/g, respectively. Statistical analysis showed that TFC observed in Aralba, Diamond, DMS and SW6237W were very similar. Regarding the total polyphenol content (TPC), the average value observed in sorghum was 2.03 mgFA/g. It is still found that PSE7431 was the genotype with the highest polyphenol content, with an average value of 2.59 mgFA/g. The lowest TPC content was observed in DMS, with an average value of 1.67 mgFA/g. The proanthocyanidins (TPAC) are oligomers and polymers of flavan-3-ol monomer units, and they have potent antioxidant activities. The role of these compounds is to protect against oxidative stress that is induced by free radicals by increasing the capacity of the cell for the absorbing of such oxygen radicals, which prevents the generation of free radicals, stimulates the detoxification enzymes, decreases lipid peroxidation, and reduces cell proliferation [50]. For this parameter, the greatest variation was observed, and five sorghum genotypes showed low TPAC levels that ranged between 0.01 mgCE/g in DMS and 0.53 mgCE/g in SW6237W. Aralba and SW6143W showed values ranging between 1.81 mgCE/g and 3.19 mgCE/g, respectively, whereas PSE7431 reached the highest content (17.07 mgCE/g). The average TPAC content of sorghum was the highest (2.93 mgCE/g) with respect to oat (1.06 mgCE/g) and maize (0.55 mgCE/g). Moreover, sorghum hybrids showed the highest TAC compared to oats (three times higher) and maize hybrids (about 23% higher), with an average content of 35.20 mmol TEAC/kg. The highest and lowest antioxidant capacity occurred in PSEAG4E44 and PSE7431, respectively, with an average value of 44.38 and 26.89 mmolTEAC/kg, respectively.

The average value of TFC differed between hulled and naked oats, being 1.27 mgCE/g and 1.08 mgCE/g, respectively, while the mean content was 1.21 mgCE/g, varying from the highest and lowest content in Abel (1.50 mgCE/g) and Kynom (0.94 mgCE/g). Our results were higher with respect to Meenu et al. [51], who found in a large collection of naked and husked oat germplasm a range that varied from 0.14 mgCE/g to 0.46 mgCE/g in naked oat, and from 0.32 mgCE/g to 0.12 mgCE/g in hulled oat. These differences in TFC could be attributable to the great interaction with the environment, as asserted by Menga et al. [52]. The average value observed for TPC was 1.49 mgFA/g, and little, but no significant differences were observed between the mean content of naked (1.51 mgFA/g) and husked (1.44 mgFA/g) genotypes. Abel was the genotype with the highest polyphenol content (1.77 mgFA/g), and Kynom and Nigra with the lowest (1.31 mgFA/g). Similarly, also in Meenu et al. [51] work, no differences were found in the TPC content between naked and husked oats. It is noteworthy that TPC measured by the Folin–Ciocalteu method may not give a full picture of the quantity and, often, of the quality of phenolic compounds present in the extracts. Scarce variability was observed in the TPAC, and the average value observed was 1.06 mgCE/g, ranging between Corneil with 1.28 mgCE/g and Genziana with 0.86 mgCE/g. No differences were observed between the mean content of naked (1.06 mgCE/g) and hulled (1.06 mgCE/g) genotypes. The highest and lowest antioxidant capacity occurred in Corneil and Kynom (13.01 mmol TE/kg and 10.48 mmol TE/kg, respectively), and the average value observed was 11.78 mmol TE/kg. The mean content of naked and hulled was 11.53 mmol TE/kg and 12.28 mmol TE/kg, respectively. The antioxidant activity measured in the samples was higher than those reported by Meenu et al. [51], but they agreed to find higher activity in the hulled genotypes compared to the naked ones. In fact, large quantities of phenolic acids and avenanthramides, which have high antioxidant capacity, were concentrated in hulls, as reported by Varga et al. [53]. In this study that investigated the scavenging capacity of hulls and groats separately, an increased antioxidant activity of hulls compared to groats was demonstrated. Oat accessions showed interesting values of total antioxidant capacity mainly due to the presence of avenanthramides. Avenanthramides are a group of phenolic compounds consisting of anthranilic acid or hydroxy anthranilic acid, and hydroxyl cinnamic acid. They are powerful antioxidants and have also been shown to have antioxidative and anti-inflammatory effects in vivo, and they are unique to oats.

Pigmented maize contains secondary metabolites, mainly represented by phenolics and carotenoids [54]. Among the polyphenols, flavonoids are recognized as a source of antioxidative, antihypertensive and anti-inflammatory activities [55]. Flavonoids are also responsible for the pericarp color, which varies from cream to blue and encompasses crimson and violet. The mean TFC in maize (7.07 mgCE/g) was about six times with respect to oat (1.21 mgCE/g) and about sixteen times with respect to sorghum (0.44 mgCE/g). The genotype with the highest flavonoid content was Kully (purple), with a value of 29.43 mgCE/g. On the contrary, Hualtaco (white) was the genotype with the lowest flavonoid content, with an average value of 1.05 mgCE/g. No significant differences for TFC were observed between the white (Hualtaco and VA116w) and yellow genotypes (VA572) (Table 3). Moreover, the range observed for those genotypes was the same as previously reported by Suriano et al. [56] of about 1.16 mgCE/g, but higher than Rodriguez-Salinas et al. [57] in a collection of native maize genotypes from Northeast Mexico. Considering the introduction of the new pigmented genotypes, VA116 purple, with an average content of 13.28 mgCE/g, showed the greatest increase (of about eleven times) in TFC compared to the parental genotype VA116w (white). Indeed, purple-colored maize genotypes (Kully and VA116w purple) had higher phenolic compounds than light- or white-colored genotypes. Regarding the TPC, a large and significant difference was observed, with an average value of 2.94 mgFA/g. Kully, the native purple Andean genotype, showed the highest content with an average value of 9.63 mgFA/g, while Hualtaco, the native white Andean genotype, had the lowest content (1.29 mgFA/g). No significant differences for TPC were observed among the white (Hualtaco, VA116w, VA522w) and the yellow genotypes (VA572). A similar range was found by Žilić et al. [54], indicating that the genotypes with purple pericarp had the highest content; on the contrary, the white and yellow genotypes that have no anthocyanin showed the lowest content. These results are consistent with our study. Moreover, the positive effect of introgression of colored genotype in Italian maize lines (VA116 purple, VA522 blue and VA572 black) has led to an increase in the TPC compared to the parents of about 60%, 25% and 55%, respectively. Great variation was observed in the TPAC (average value of 0.55 mgCE/g), ranging between Kully (2.40 mgCE/g) and VA116w (0.06 mgCE/g). No significant differences were observed among the white-type genotypes (Hualtaco, VA116w, VA522w) and the yellow genotype (VA572). Our results were in accordance with the study of Suriano et al. [56] and Rodriguez-Salinas et al. [57]. The TAC measured by ABTS assay had an average content of 28.66 mmolTE/kg. Compared to sorghum, which has an average value of 35.20 mmolTE/kg but with rather homogeneous values between genotypes, greater variability was highlighted in maize. In this regard, the highest TAC was detected in Kully and VA116, the purple genotypes, with values of 89.16 and 40.83 mmolTE/kg, respectively. The lowest activity was observed in VA572 (10.38 mmolTE/kg), which was not statistically different from VA116w and VA522w, the white and yellow genotypes. Also, for this parameter, the introgression of colored genotype in Italian maize lines led to an increase in TAC compared to the parents (69%, 43%, and 75%, respectively). The two Bolivian genotypes Kully (purple) and Hualtaco (white) showed opposite antioxidant traits, with Kully having the highest levels of TFC, TPC, TPAC and TAC while Hualtaco had the lowest values. Therefore, in accordance with the literature, our data confirm that a darker-colored maize kernel had more antioxidant activity [54].

#### 3.1.7. Correlations among the Different Investigated Traits

For a better understanding of the pasting properties, it must be highlighted that they are related to the amylose content. A high amylose content limits starch swelling by forming complexes with lipids, which in turn results in a lower peak viscosity and setback [58]. In sorghum, this feature occurs, and a negative correlation was found between amylose and PT (*p* ≤ 0.01 r = −0786). This phenomenon is explained by the low solubility of the amylose molecule compared to amylopectin, which in turn produces the effect of lowering the temperature required for gelatinization. Accordingly, the PT provides an indication of the minimum temperature required to cook flour [59]. The same was not so clearly observed for oat genotypes because the high content of beta-glucan, acting as a hydrocolloid, contributes to increasing the viscosity [60]. Starch gelatinization was important for its capacity to form a matrix in which the gas bubbles are entrapped, producing a crucial impact in the gluten-free formulation of bakery products. According to Abdel-Aal [61], the addition of gel-forming starches (for example, pregelatinized starches) is a useful means to provide gas blockage as a stabilizing agent. Moreover, a negative and significant correlation was detected with a setback for oat and sorghum (*p* ≤ 0.05 r = −0.573 and −0.608, respectively), as previously indicated by Acquistucci et al. [62]. Thus, flours with high amylose content develop low viscosity after heating and a low re-organization rate after the cooling period, providing a low final viscosity. Retrogradation is linked to the increase in the consistency or hardness of the starch [63], and in baking, this process causes the undesirable staling process of bread during storage [64]. In GF pasta production, instead, all flours that are more susceptible to gelatinization (PV) and retrogradation (setback) are preferable [62].

Sorghum showed a significant and negative correlation between fiber content and peak temperature, PV and setback (*p* ≤ 0.05 r = −0.614, −0.556 and −0.525, respectively). This result is consistent because the fiber interferes with the gelatinization of the starch, causing a reduction in the parameters related to viscosity [65]. For oats, however, a positive correlation with the peak temperature is observed (*p* ≤ 0.05 r = 0.493), probably due to the specific composition of the fiber, namely high beta-glucan content.

In maize, few significant correlations were found with resistant starch (negative correlation with PV, *p* ≤ 0.05 r = −0.553), total starch (negative correlation with PT and setback, *p* ≤ 0.05 r = −0.560 and −0.550, respectively) and none with amylose. Instead, several significant correlations between fiber and protein were found. Peak temperature and pasting temperature positively correlated with fiber (*p* ≤ 0.05, r = 0.534 and 0.525, respectively) and proteins (*p* ≤ 0.01 r = 0.885 and 0.960, respectively). The positive correlation between proteins and fiber with pasting temperature could be explained by the effect produced by these latter in delaying the gelation of amylose. Conversely, PV and BD negatively correlated with proteins and fiber (*p* ≤ 0.05, r = −0.520 and −0.721 for proteins and r = −0.553 and −0.547 for fiber, respectively), as previously observed by Sandhu and Singh [59], because they form complex with amylose that in turn caused the decrease of viscosity (PV) and BD.

Regarding the total antioxidant capacity in maize, significant correlations were observed with TPC, TFC, and TPAC (*p* ≤ 0.01, r = 0.982, 0.987 and 0.984, respectively) (On the contrary, in sorghum, only negative and significant correlations were found between TAC and TPAC (*p* ≤ 0.05 r = −0.580), whereas in oat, a significant correlation was observed only with TPC (*p* = 0.05, r = 0.482). These features suggest that in maize, the analyzed compounds contributed the most to TAC, whereas in the other two species, other bioactive compounds impacted the TAC levels. Moreover, a fair but significant correlation with β-glucan in oats (*p* ≤ 0.01, r = 0.601) suggested that the antioxidant capacity correlated with this soluble fiber component, located in the outermost part of the kernel [66].

## 4. Conclusions

According to the results of this research, we can assume that all three species possess technological indexes suitable for food processing. Moreover, among oats and maize, there are interesting nutritional components that could be desirable in food formulation for improving health. Our results clearly indicated that based on the type of food to be formulated, some identified genotypes present adequate characteristics for the formulation of GF pasta or bakery products. In this regard, among oat genotypes, Terra and Lexic could be interesting for GF pasta with a high value of Setback; conversely, Kripton showing a lower retrogradation degree could be more apt for baking goods. Among sorghum genotypes, SW6129 and SW6237W, which have the highest PV and Setback, could be appreciable for food products that request stable thickening after heating and cooling, like in sauces production, while PSEAG4E44 with low PV and setback, is more suitable for baking goods. For maize, VA572 black could be interesting for its low BD and setback and is suitable for baking goods, while Kully and VA116Wpurple are the most interesting for TAC. Therefore, the adequate blending of whole grain flours from the different GF cereals presented in this study could allow the manufacturing of products of good technological and healthier nutritional quality. Furthermore, the great challenge of the nutritional and sensorial aspects of GF products must be carefully evaluated. Finally, three different GXE trials are ongoing in Northern, Central and Southern Italian Regions, using the genotypes abovementioned of each species, selected for the most promising traits in terms of technological and nutritional performance. These multi-environmental trails will allow us to identify the effect of the area and the year of cultivation on the variability of the nutritional and pasting properties of the three cereal species.

## Figures and Tables

**Table 1:** Technological and nutritional traits of the twenty-six genotypes of sorghum, oat and maize.

SAMPLES		TKW		Protein		TDF		β-Glucan		RS		TS		Amylose	
		(g)		(%)		(%)		(%)		(%)		(%)		(%)	
		Mean	sd	Mean	sd	Mean	sd	Mean	sd	Mean	sd	Mean	sd	Mean	sd
SORGHUM	Aralba	24.33 ^ab^	1.82	10.62 ^c^	0.19	9.40 ^c^	0.55	n.d.		0.48 ^e^	0.05	69.49 ^c^	0.35	23.90 ^b^	0.87
	Diamond	23.76 ^ac^	0.20	12.68 ^a^	0.04	10.89 ^c^	0.21	n.d.		0.40 ^e^	0.01	57.77 ^f^	0.98	26.51 ^b^	3.28
	DMS	25.62 ^a^	0.20	11.56 ^b^	0.03	11.41 ^b^	0.22	n.d.		0.44 ^e^	0.00	73.04 ^ab^	0.97	26.56 ^b^	3.18
	PSEAG4E44	22.00 ^bc^	0.50	9.44 ^ef^	0.18	12.21 ^a^	0.18	n.d.		0.51 ^e^	0.02	65.57 ^e^	1.49	54.40 ^a^	2.84
	PSE7431	21.30 ^c^	2.30	9.80 ^de^	0.11	11.75 ^ab^	0.06	n.d.		0.66 ^d^	0.05	67.02 ^de^	1.11	16.83 ^c^	1.21
	SW6129	22.93 ^bc^	0.70	9.20 ^f^	0.36	8.52 ^f^	0.33	n.d.		2.32 ^a^	0.10	69.19 ^cd^	1.18	27.37 ^b^	0.92
	SW6143W	22.7 0 ^bc^	1.50	10.91 ^c^	0.46	10.22 ^d^	0.40	n.d.		1.48 ^c^	0.09	72.22 ^b^	1.38	15.64 ^c^	1.74
	SW6237W	23.47 ^abc^	1.72	9.90 ^d^	0.39	8.23 ^f^	0.66	n.d.		2.02 ^b^	0.12	74.88 ^a^	0.12	25.75 ^b^	4.79
	Means ± SE	23.26 ± 1.72		10.51 ± 1.15		10.33 ± 1.44				1.04 ± 0.75		68.65 ± 5.18		27.12 ± 11.68	
OAT	Abel	15.60 ^f^	0.42	13.42 ^e^	0.06	9.84 ^ce^	0.33	3.97 ^b^	0.10	0.51 ^bc^	0.00	51.65 ^d^	0.25	17.11 ^c^	0.44
	Corneil	19.75 ^c^	0.07	17.00 ^a^	0.00	10.11 ^cd^	1.21	4.63 ^a^	0.16	0.52 ^b^	0.00	53.58 ^cd^	1.24	16.64 ^c^	0.23
	Genziana	23.70 ^a^	0.14	13.18 ^f^	0.03	10.03 ^ce^	0.03	3.07 ^cd^	0.59	0.51 ^bc^	0.00	60.67 ^a^	0.83	15.37 ^c^	1.70
	Konradin	20.10 ^c^	0.28	13.65 ^d^	0.02	9.75 ^ce^	0.39	3.07 ^cd^	0.25	0.48 ^de^	0.00	61.38 ^a^	1.76	16.45 ^c^	1.72
	Kripton	12.55 ^g^	0.07	13.55 ^de^	0.02	11.38 ^b^	0.01	3.01 ^cd^	0.21	0.53 ^b^	0.01	59.96 ^ab^	1.02	28.98 ^b^	1.01
	Kynom	18.55 ^d^	0.07	13.71 ^d^	0.03	8.89 ^e^	0.34	2.52 ^d^	0.11	0.47 ^e^	0.00	56.57 ^bc^	3.66	8.88 ^d^	0.59
	Lexic	16.45 ^e^	0.07	13.40 ^e^	0.06	8.91 ^de^	0.96	3.18 ^c^	0.04	0.55 ^a^	0.01	56.94 ^bc^	0.97	7.08 ^d^	1.13
	Nigra	21.90 ^b^	0.14	14.17 ^c^	0.13	10.79 ^bc^	0.44	2.76 ^cd^	0.13	0.50 ^cd^	0.01	58.91 ^ab^	1.34	32.78 ^a^	5.01
	Terra	15.70 ^f^	0.14	14.52 ^b^	0.17	14.25 ^a^	0.20	4.80 ^a^	0.23	0.48 ^de^	0.00	55.16 ^c^	1.08	7.12 ^d^	1.43
	Means ± SE	18.26 ± 3.40		14.07 ± 1.14		10.44 ± 1.64		3.45 ±0.82		0.50 ± 0.03		57.20 ± 3.44		16.71 ± 8.91	
MAIZE	Hualtaco	741.50 ^a^	2.1	6.93 ^i^	0.13	8.19 ^b^	0.88	n.d.		0.08 ^b^	0.01	70.91^a^	0.01	11.27 ^b^	2.58
	Kully	322.00 ^d^	1.4	7.98 ^h^	0.14	9.77 ^a^	0.34	n.d.		0.21 ^a^	0.07	70.67 ^a^	1.12	10.72 ^b^	2.95
	VA 116 W	246.00 ^h^	1.4	10.99 ^e^	0.06	10.90 ^a^	0.39	n.d.		0.15 ^ab^	0.01	64.34 ^c^	0.69	21.64 ^a^	0.89
	VA 116 W Purple	305.50 ^e^	2.1	13.35 ^a^	0.06	10.16 ^a^	0.33	n.d.		0.24 ^a^	0.07	66.65 ^bc^	2.22	8.26 ^b^	0.32
	VA 1268 Red	421.50 ^b^	2.1	11.87 ^d^	0.12	10.60 ^a^	0.66	n.d.		0.18 ^a^	0.01	67.00 ^bc^	0.83	6.33 ^b^	0.68
	VA 522 W	355.50 ^c^	2.1	13.01 ^b^	0.10	9.43 ^ab^	0.42	n.d.		0.17 ^ab^	0.06	66.74 ^bc^	0.04	10.35 ^b^	3.46
	VA 522 W Blue	422.50 ^b^	2.1	10.01 ^f^	0.08	8.12 ^b^	1.19	n.d.		0.23 ^a^	0.02	71.09 ^a^	2.17	10.30 ^b^	1.66
	VA 572	286.50 ^f^	2.1	12.34 ^c^	0.05	10.70 ^a^	0.64	n.d.		0.17 ^ab^	0.01	69.18 ^ab^	2.31	8.61 ^b^	1.08
	VA 572 Black	280.00 ^g^	1.4	9.38 ^g^	0.10	10.32 ^a^	0.09	n.d.		0.24 ^a^	0.02	71.64 ^a^	2.62	7.80 ^b^	1.81
	Means ± SE	375.70 ± 145.6		10.70 ± 2.19		9.80 ± 1.11				0.18 ± 0.06		68.69 ± 2.77		10.59 ± 4.55	

Results presented in the table are expressed as the mean value and standard deviation (SD) for three replications. Different letters in the same column indicate significant differences at *p* ≤ 0.05. sd = standard deviation; SE = standard error of mean; TKW = Thousand kernel weight; TDF = Total Dietary Fibre; RS = Resistant Starch; TS = Total Starch; n.d. = not determined.

**Table 2:** Micro Visco Analyzer parameters of the twenty-six genotypes of sorghum, oat and maize.

SAMPLES		Pasting Temperature		Peak Viscosity		Breakdown		Setback	
		(°C)		(BU)		(BU)		(BU)	
		Mean	sd	Mean	sd	Mean	sd	Mean	sd
SORGHUM	Aralba	76.50 ^a^	0.57	754.50 ^ab^	130.8	421.50 ^b^	115.3	671.50 ^cd^	67.2
	Diamond	76.45 ^a^	0.49	822.50 ^a^	40.3	435.50 ^b^	70.0	687.50 ^bc^	34.6
	DMS	76.90 ^a^	0.00	766.00 ^ab^	69.3	431.50 ^b^	71.4	646.50 ^d^	61.5
	PSEAG4E44	65.20 ^d^	0.14	697.50 ^b^	136.5	505.00 ^a^	121.6	611.50 ^e^	43.1
	PSE7431	73.50 ^c^	0.28	512.50 ^c^	98.3	350.50 ^c^	72.8	699.50 ^abc^	64.3
	SW6129	74.85 ^b^	0.07	808.00 ^a^	90.5	406.00 ^b^	99.0	706.50 ^abc^	27.6
	SW6143W	74.75 ^b^	0.35	768.00 ^ab^	91.9	407.00 ^b^	84.9	725.00 ^a^	31.1
	SW6237W	74.85 ^b^	0.92	813.00 ^a^	80.6	402.50 ^b^	84.1	708.50 ^ab^	44.5
	Means ± SE	74.13 ± 3.67		742.80 ± 120.8		419.90 ± 79.0		682.10 ± 50.9	
OAT	Abel	61.56 ^ab^	0.76	595.00 ^cd^	7.1	404.00 ^bc^	5.7	710.00 ^ab^	7.1
	Corneil	58.65 ^bc^	1.20	617.00 ^cd^	8.5	423.00 ^b^	24.0	705.50 ^ab^	43.1
	Genziana	50.65 ^d^	3.75	932.50 ^a^	46.0	548.00 ^a^	32.5	671.50 ^ab^	60.1
	Konradin	62.25 ^ab^	0.21	591.50 ^cd^	87.0	353.00 ^bc^	60.8	637.00 ^b^	53.7
	Kripton	66.15 ^a^	0.35	752.00 ^b^	53.7	429.00 ^b^	17.0	588.50 ^b^	48.8
	Kynom	65.45 ^a^	1.91	683.00 ^bc^	33.9	372.00 ^bc^	41.0	646.50 ^ab^	40.3
	Lexic	58.55 ^bc^	1.20	532.50 ^d^	37.5	333.50 ^c^	48.8	802.00 ^a^	137.2
	Nigra	54.45 ^cd^	1.77	782.50 ^b^	0.7	431.00 ^b^	8.5	656.50 ^ab^	46.0
	Terra	62.90 ^ab^	5.66	687.50 ^bc^	60.1	375.50 ^bc^	20.5	798.50 ^a^	34.6
	Means ± SE	60.07 ± 5.24		685.90 ± 123.4		407.70 ± 65.8		690.70 ± 83.1	
MAIZE	Hualtaco	67.15 ^d^	1.20	1204.5 ^a^	101.1	708.00 ^a^	75.0	439.50 ^b^	245.4
	Kully	67.25 ^d^	1.48	736.5 ^bd^	115.3	498.00 ^b^	70.7	588.50 ^ab^	4.9
	VA 116 W	72.45 ^b^	0.49	784.5 ^bc^	34.6	411.00 ^bc^	32.5	683.50 ^ab^	36.1
	VA 116 W Purple	74.55 ^a^	0.21	666.5 ^ce^	30.4	342.00 ^ce^	21.2	684.00 ^ab^	125.9
	VA 1268 Red	73.50 ^ab^	0.42	735.5 ^bd^	57.3	369.00 ^cd^	52.3	646.00 ^ab^	38.2
	VA 522 W	73.45 ^ab^	0.35	633.5 ^de^	72.8	268.50 ^df^	61.5	670.50 ^ab^	88.4
	VA 522 W Blue	70.65 ^c^	0.49	837.0 ^b^	59.4	420.00 ^bc^	8.5	647.00 ^ab^	38.2
	VA 572	74.50 ^a^	0.28	553.5 ^e^	57.3	203.00 ^f^	25.5	715.00 ^a^	29.7
	VA 572 Black	69.75 ^c^	1.06	412.5 ^f^	12.0	237.00 ^ef^	15.6	472.00 ^ab^	11.3
	Means ± SE	71.47 ± 2.88		729.3 ± 218.4		384.10 ± 153.2		616.20 ± 119.3	

Results presented in the table are expressed as the mean value and standard deviation (SD) for three replications. Different letters in the same column indicate significant differences at *p* ≤ 0.05. sd = standard deviation; SE = standard error of mean; BU = Brabender Unit.

**Table 3:** Total flavonoid, phenolic, proanthocyanidin contents and antioxidant capacities of the twenty-six genotypes of sorghum, oat and maize.

SAMPLES		TFC		TPC		TPAC		TAC (ABTS)	
		(mgCE/g)		(mgFA/g)		(mgCE/g)		(mmol TE/Kg)	
		Mean	sd	Mean	sd	Mean	sd	Mean	sd
SORGHUM	Aralba	0.35 ^cd^	0.01	1.87 ^cd^	0.07	1.81 ^c^	0.01	31.66 ^cd^	0.90
	Diamond	0.33 ^cd^	0.00	1.77 ^de^	0.04	0.11 ^f^	0.03	30.57 ^d^	0.01
	DMS	0.34 ^cd^	0.03	1.67 ^e^	0.02	0.01 ^f^	0.01	34.68 ^c^	1.16
	PSEAG4E44	0.45 ^bc^	0.01	2.26 ^b^	0.04	0.67 ^d^	0.11	44.38 ^a^	2.71
	PSE7431	0.85 ^a^	0.02	2.59 ^a^	0.17	17.07 ^a^	0.02	26.89 ^e^	2.07
	SW6129	0.31 ^d^	0.04	1.70 ^de^	0.04	0.08 ^f^	0.03	43.77 ^a^	0.13
	SW6143W	0.54 ^b^	0.17	2.41 ^b^	0.08	3.19 ^b^	0.02	31.53 ^cd^	0.39
	SW6237W	0.38 ^cd^	0.04	1.96 ^c^	0.01	0.53 ^e^	0.02	38.15 ^b^	1.03
	Means ± SE	0.44 ± 0.18		2.03 ± 0.33		2.93 ± 5.56		35.20 ± 6.13	
OAT	Abel	1.50 ^a^	0.00	1.77 ^a^	0.06	0.99 ^bc^	0.04	11.94 ^b^	0.32
	Corneil	0.97 ^fg^	0.12	1.62 ^b^	0.04	1.28 ^a^	0.07	13.01 ^a^	0.09
	Genziana	1.22 ^cd^	0.08	1.39 ^cd^	0.02	0.86 ^c^	0.11	12.19 ^b^	0.04
	Konradin	1.30 ^bc^	0.04	1.46 ^c^	0.05	1.17 ^ab^	0.08	11.65 ^b^	0.37
	Kripton	1.11 ^de^	0.00	1.42 ^c^	0.05	1.01 ^bc^	0.01	11.69 ^b^	0.43
	Kynom	0.94 ^g^	0.08	1.31 ^d^	0.03	1.04 ^bc^	0.18	10.48 ^c^	0.21
	Lexic	1.42 ^ab^	0.04	1.57 ^b^	0.00	1.03 ^bc^	0.11	11.65 ^b^	0.13
	Nigra	1.08 ^ef^	0.04	1.31 ^d^	0.03	1.04 ^bc^	0.05	11.65 ^b^	0.23
	Terra	1.36 ^b^	0.04	1.56 ^b^	0.05	1.11 ^ab^	0.00	11.78 ^b^	0.12
	Means ± SE	1.21 ± 0.20		1.49 ± 0.15		1.06 ± 0.13		11.78 ± 0.66	
MAIZE	Hualtaco	1.05 ^g^	0.16	1.29 ^f^	0.01	0.18 ^d^	0.01	13.32 ^de^	0.68
	Kully	29.43 ^a^	0.28	9.63 ^a^	0.18	2.40 ^a^	0.19	89.16 ^a^	7.90
	VA 116 W	1.19 ^g^	0.12	1.54 ^f^	0.03	0.06 ^d^	0.00	12.72 ^e^	0.51
	VA 116 W Purple	13.28 ^b^	0.20	3.83 ^b^	0.22	0.61 ^c^	0.04	40.83 ^b^	0.06
	VA 1268 Red	3.79 ^d^	0.04	2.22 ^d^	0.03	0.77 ^b^	0.06	19.80 ^c^	0.22
	VA 522 W	1.70 ^f^	0.04	1.37 ^f^	0.11	0.14 ^d^	0.02	10.80 ^e^	0.20
	VA 522 W Blue	2.35 ^e^	0.16	1.85 ^e^	0.04	0.12 ^d^	0.02	19.14 ^cd^	2.13
	VA 572	1.28 ^g^	0.00	1.45 ^f^	0.04	0.08 ^d^	0.00	10.38 ^e^	0.16
	VA 572 Black	9.55 ^c^	0.12	3.27 ^c^	0.12	0.59 ^c^	0.05	41.79 ^b^	1.92
	Means ± SE	7.07 ± 9.14		2.94 ± 2.59		0.55 ± 0.72		28.66 ± 25.04	

Results presented in the table are expressed as the mean value and standard deviation (SD) for three replications. Different letters in the same column indicate significant differences at *p* ≤ 0.05. sd = standard deviation; SE = standard error of mean; TFC = total flavonoid content; TPC = total phenolic content; TPAC = total proanthocyanidin content; TAC = total antioxidant activity; TE = Trolox equivalent; FA = ferulic acid equivalent; CE = catechin equivalent.

## Data Availability

The original contributions presented in the study are included in the article, further inquiries can be directed to the corresponding author.

## References

[1] See J.A., Kaukinen K., Makharia G.K., Gibson P.R., Murray J.A. (2015). Practical insights into gluten-free diets. Nat. Rev. Gastroenterol. Hepatol..

[2] Tortora R., Capone P., De Stefano G., Imperatore N., Gerbino N., Donetto S., Monaco V., Caporaso N., Rispo A. (2015). Metabolic syndrome in patients with coeliac disease on a gluten-free diet. Aliment. Pharmacol. Ther..

[3] Picascia A., Camarca M., Malamisura R., Mandile M., Galatola D. (2020). In celiac disease patients the in vivo challenge with the diploid Triticum monococcum elicits a reduced immune response compared to hexaploid wheat. Mol. Nutr. Food Res..

[4] Bongianino N.F., Steffolani M.E., Morales C.D., Biasutti C.A., León A.E. (2023). Technological and Sensory Quality of Gluten-Free Pasta Made from Flint Maize Cultivars. Foods.

[5] Kulamarva A.G., Venkatesh R.S., Raghavan V.G.S. (2009). Nutritional and Rheological Properties of Sorghum. Int. J. Food Prop..

[6] Del Giudice F., Massardo D.R., Pontieri P., Maddaluno L., De Vita P., Fares C., Ciacci C., Del Giudice L. (2008). Development of a sorghum chain in the Italian Campania Region: From the field to the celiac patient’s table. J. Plant Interact..

[7] Aguiar E.V., Santos F.G., Queiroz V.A.V., Capriles V.D. (2023). A Decade of Evidence of Sorghum Potential in the Development of Novel Food Products: Insights from a Bibliometric Analysis. Foods.

[8] Xu F., Dube N.M., Han, Zhao R., Wang Y., Chen J. (2021). The Effect of Zimbabwean Tannin-Free Sorghum Flour Substitution on Fine Dried Noodles Quality Characteristics. J. Cereal Sci..

[9] Galassi E., Gazza L., Nocente F., Kouagang Tchakoutio P., Natale C., Taddei F. (2023). Valorization of two African typical crops, Sorghum and Cassava, by the production of different dry pasta formulations. Plants.

[10] Bvenura C., Kambizi L., Rajeev B. (2022). Chapter 5—Future Grain Crops. Future Foods.

[11] Peterson D.M. (2001). Oat Antioxidants. J. Cereal Sci..

[12] Associazione Italiana Celiachia (AIC). https://www.celiachia.it/dieta-senza-glutine/gestire-gluten-free-diet/cereals-allowed/.

[13] Elfström P., Sundström J., Ludvigsson J.F. (2014). Systematic review with meta-analysis: Associations between coeliac disease and type 1 diabetes. Aliment. Pharmacol. Ther..

[14] Smyth D.J., Plagnol V., Walker N.M., Cooper J.D., Downes K., Yang J.H., Howson J.M., Stevens H., McManus R., Wijmenga C. (2008). Shared and distinct genetic variants in type 1 diabetes and celiac disease. N. Engl. Med..

[15] Rooney L.W., Serna Saldívar S.O., White P.J., Johnson L.A. (2003). Food use of whole corn and dry-milled fractions. Corn: Chemistry and Technology.

[16] Nuss E.T., Tanumihardjo S.A. (2010). Maize: A paramount staple crop in the context of global nutrition. Food Sci. Food Saf..

[17] Hu Q.P., Xu J.G. (2011). Profiles of carotenoids, anthocyanins, phenolics and antioxidant activity of selected color waxy corn grains during maturation. J. Agr. Food Chem..

[18] Zillić S., Serpen A., Akillioĝlu G., Vanĉetović V. (2012). Phenolic compounds, carotenoids, anthocyanins and antioxidant capacity of colored maize (*Zea mays* L.) kernel. J. Agric. Food Chem..

[19] Li Q., Somavat P., Singh V., Chatham L., Mejia E.G. (2017). A comparative study of anthocyanins distribution in purple and red corn coproducts from three conventional fractionation process. Food Chem..

[20] Menga V., Amato M., Phillips T.D., Angelino D., Morreale F., Fares C. (2017). Gluten-free pasta incorporating chia (*Salvia hispanica* L.) as thickening agent: An approach to naturally improve the nutritional profile and the in vitro carbohydrate digestibility. Food Chem..

[21] International Association for Cereal Science and Technology (2003). ICC Standard Methods (Methods No. 105/2).

[22] Mariotti F., Tomé D., Mirand P.P. (2008). Converting nitrogen into protein—Beyond 6.25 and Jones’ factors. Crit. Rev. Food Sci. Nutr..

[23] Association of Official Analytical Chemists (2005). Official Methods of Analysis 996.11.

[24] Association of Official Analytical Chemists (2002). Official Methods of Analysis 2002.02, Resistant Starch in Starch and Plant Materials.

[25] Cunniff P., Association of Official Analytical Chemists (1995). Official Methods of Analysis 991.

[26] (2010). Cereals and Pulses-Determination of the Mass of 1000 Grains.

[27] (2009). Determination of Bulk Density, Called Mass per Hectolitre-Part 1: Reference Method.

[28] McCleary B.V., Mugford D.C., Camire M.C., Gibson T.S., Harrigan K., Janning M., Meuser F., Williams P. (1997). Determination of ß-glucan in barley and oats by streamlined enzymatic method: Summary of collaborative study. J. AOAC Int..

[29] Beta T., Nam S., Dexter J.E., Sapirstein H.D. (2005). Phenolic content and antioxidant activity of pearled wheat and roller-milled fractions. Cereal Chem..

[30] Singleton V.L., Rossi J.A. (1965). Colorimetry of Total Phenolics with Phosphomolybdic-Phosphotungstic Acid Reagents. Am. J. Enol. Vitic..

[31] Sun B., da-Silva J.M.R., Spranger I. (1998). Critical Factors of Vanillin Assay for Catechins and Proanthocyanidins. J. Agric. Food Chem..

[32] Eberhardt M.V., Lee C.Y., Liu R.H. (2000). Antioxidant activity of fresh apples. Nature.

[33] Re R., Pellegrini N., Proteggente A., Pannala A., Yang M., Rice-Evans C. (1999). Antioxidant activity applying an improved ABTS radical cation decolorization assay. Free Radic. Biol. Med..

[34] Marengo M., Barbiroli A., Bonomi F., Casiraghi M.C., Marti A., Pagani M.A., Manful J., Graham-Acquaah S., Ragg E., Fessas D. (2017). Macromolecular Traits in the African Rice *Oryza glaberrima* and in Glaberrima/Sativa Crosses, and Their Relevance to Processing. J. Food Sci..

[35] Wang K., Fu B.X. (2020). Inter-Relationships between Test Weight, Thousand Kernel Weight, Kernel Size Distribution and Their Effects on Durum Wheat Milling, Semolina Composition and Pasta Processing Quality. Foods.

[36] Galassi E., Taddei F., Ciccoritti R., Nocente F., Gazza L. (2020). Biochemical and technological characterization of two C4 gluten-free cereals: *Sorghum bicolor* and *Eragrostis tef*. Cereal Chem..

[37] Aruna O.A., Oluwaseyi I.O., Babatunde S.E., Adeniyi O. (2020). Effects of Different Rates of Poultry Manure and Split Applications of Urea Fertilizer on Soil Chemical Properties, Growth, and Yield of Maize. Sci. World J..

[38] Buerstmayr H., Krenn N., Stephan U., Grausgruber H., Zechner E. (2007). Agronomic performance and quality of oat (*Avena sativa* L.) genotypes of worldwide origin produced under Central European growing conditions. Field Crops Res..

[39] Korus J., Chmielewska A., Witczak M., Ziobro R., Juszczak L. (2021). Rapeseed protein as a novel ingredient of gluten-free bread. Eur. Food Res. Technol..

[40] Ziobro R., Juszczak L., Witczak M., Korus J. (2016). Non-gluten proteins as structure forming agents in gluten free bread. J. Food Sci. Technol..

[41] Lee A.R., Ng D.L., Dave E., Ciaccio E.J., Green P.H. (2009). The effect of substituting alternative grains in the diet on the nutritional profile of the gluten-free diet. J. Hum. Nutr. Diet..

[42] Blandino M., Alfieri M., Giordano D., Vanara F., Redaelli R. (2017). Distribution of bioactive compounds in maize fractions obtained in two different types of large-scale milling processes. J. Cereal Sci..

[43] Wang Q., Ellis P.R. (2014). Oat β-glucan: Physico-chemical characteristics in relation to its blood-glucose and cholesterol-lowering properties. Br. J. Nutr..

[44] Bojarczuk A., Skąpska S., Khaneghah A.M., Marszałek K. (2022). Health benefits of resistant starch: A review of the literature. J. Funct. Foods.

[45] Biselli C., Volante A., Desiderio F., Tondelli A., Gianinetti A., Finocchiaro F., Taddei F., Gazza L., Sgrulletta D., Cattivelli L. (2019). GWAS for Starch-Related Parameters in Japonica Rice (*Oryza sativa* L.). Plants.

[46] Annison G., Topping D.L. (1994). Nutritional role of resistant starch: Chemical structure vs physiological function. Ann. Rev. Nutr..

[47] Cozzolino D. (2016). The use of the rapid visco analyser (RVA) in breeding and selection of cereals. J. Cereal Sci..

[48] Batey I.L., Crosbie G.B., Ross A.S. (2007). Interpretation of RVA curves. The RVA Handbook.

[49] Alfieri M., Bresciani A., Zanoletti M., Pagani M.A., Marti A., Redaelli R. (2020). Physical, chemical and pasting features of maize Italian inbred lines. Eur. Food Res. Tech.

[50] Wang L.S., Stoner G.D. (2008). Anthocyanins and their role in cancer prevention. Cancer Lett..

[51] Meenu M., Cozzolino D., Xu B. (2023). Non-destructive prediction of total phenolics and antioxidants in hulled and naked oat genotypes with near-infrared spectroscopy. J. Food Meas. Charact..

[52] Menga V., Fares C., Troccoli A., Cattivelli L., Baiano A. (2009). Effects of genotype, location and baking on the phenolic content and some antioxidant properties of cereal species. Int. J. Food Sci. Tech.

[53] Varga M., Jójárt R., Fónad P., Mihály R., Palágyi A. (2018). Phenolic composition and antioxidant activity of colored oat. Food Chem..

[54] Žilić S., Serpen A., Akıllıoğlu G., Janković M., Gökmen V. (2012). Distributions of phenolic compounds, yellow pigments and oxidative enzymes in wheat grains and their relation to antioxidant capacity of bran and debranned flour. J. Cereal Sci..

[55] Zielińska D., Turemko M., Kwiatkowski J., Zieliński H. (2012). Evaluation of Flavonoid Contents and Antioxidant Capacity of the Aerial Parts of Common and Tartary Buckwheat Plants. Molecules.

[56] Suriano S., Balconi C., Valoti P., Redaelli R. (2021). Comparison of total polyphenol, profile anthocyanins, color analysis, carotenoids and tocols in pigmented maize. Food Sci. Tech.

[57] Rodriguez-Salinas P.A., Zavala-Garcia F., Urias-Orona V., Muy-Rangel D., Heredia J.B., Nino-Medina G. (2019). Chromatic, Nutritional and Nutraceutical Properties of Pigmented Native Maize (*Zea mays* L.) Genotypes from the Northeast of Mexico. Arab. J. Sci. Eng..

[58] Sang Y., Scott B., Seib P.A., Pedersen J., Shi Y.C. (2008). Structure and functional properties of Sorghum starch differing in amylose content. J. Agric. Food Chem..

[59] Sandhu K.S., Singh Malhi N. (2007). Some properties of corn grains and their flours I: Physicochemical, functional and chapatti-making properties of flours. Food Chem..

[60] Karp S., Wyrwisz J., Kurek M.A. (2020). The impact of different levels of oat β-glucan and water on gluten-free cake rheology and physicochemical characterisation. J. Food Sci. Technol..

[61] Abdel-Aal E.S.M., Gallagher E. (2009). Functionality of starches and hydrocolloids in gluten-free foods. Gluten-Free Food Science Technology.

[62] Acquistucci R., Francisci R., Bucci R., Ritota M., Mazzini F. (2009). Nutritional and physicochemical characterization of Italian rice flours starches. Food Sci. Tech Res..

[63] Shrestha A.K., Halley P.J., Halley P.J., Avérous L.R. (2014). Starch modification to develop novel starch-biopolymer blends: State of art and perspectives. Starch Polymers: From Genetic Engineering to Green Applications.

[64] Gray J., Bemiller J. (2003). Bread staling: Molecular basis and control. Compr. Rev. Food Sci. Food Saf..

[65] Laguna L., Sanz T., Sahi S., Fiszman S.M. (2014). Role of fibre morphology in some quality features of fibre-enriched biscuits. Int. J. Food Prop..

[66] Emmons C.L., Peterson D.M., Paul G.L. (1999). Antioxidant capacity of oat (*Avena sativa* L.) extracts. 2. In vitro antioxidant activity and contents of phenolic and tocol antioxidants. J. Agric. Food Chem..

